# RACE AND GENDER DIFFERENCES IN PHYSICAL FUNCTIONING AMONG COMMUNITY-DWELLING OLDER ADULTS

**DOI:** 10.1093/geroni/igz038.2868

**Published:** 2019-11-08

**Authors:** Chanee D Fabius, Lauren J Parker, Roland J Thorpe Jr.

**Affiliations:** Johns Hopkins University, Baltimore, Maryland, United States

## Abstract

Prior work has demonstrated that there are race and gender disparities in the prevalence of need for assistance with tasks such as self-care, mobility, and household activities. Research has historically shown that older black Americans and women experience greater prevalence of physical functioning declines. It is unclear whether these differences persist among those receiving assistance. Using data from the 2015 National Health and Aging Trends Study (NHATS), a nationally representative study of Medicare beneficiaries aged 65 and older, and after adjusting for covariates, black men received less assistance with self-care and mobility activities, and white and black women received more help with mobility and household activities, compared to white men. Findings are critical to advancing our understanding of the needs of vulnerable older adults receiving assistance. More research is needed to understand the implications of these differences on long-term services and supports provided by both informal and formal caregivers.

